# Attentional processing of body images in women with overweight and obesity

**DOI:** 10.1007/s40519-022-01419-1

**Published:** 2022-07-04

**Authors:** Julia Baur, Kerstin Krohmer, Eva Naumann, Jennifer Svaldi

**Affiliations:** Department of Clinical Psychology and Psychotherapy, University of Tuebingen, Schleichstraße 4, 72076 Tübingen, Germany

**Keywords:** Obesity, Overweight, Attentional bias, Eye-tracking, Body image

## Abstract

**Purpose:**

Despite the claim to integrate body image interventions in obesity treatment, little is known about the mechanisms involved in maintaining body dissatisfaction in persons with overweight and obesity. Therefore, the present study sought to investigate attentional processing of body stimuli in women with overweight and obesity (OW).

**Methods:**

Women with OW (*n* = 82) and normal weight controls (NW; *n* = 44) conducted two eye-tracking paradigms. In the first paradigm, fixation duration on the subjectively most beautiful and ugliest body part of one’s own and a weight-matched control body were analyzed. In the second paradigm, picture pairs including the own and a control body or object were presented and initial fixation orientation was measured. Automatic and intentional processing of the body pictures was manipulated by either indicating on which side which stimuli would appear or not.

**Results:**

Women with OW displayed a bias towards the ugliest as opposed to the most beautiful body part, whereas women with NW showed a balanced viewing pattern. Furthermore, both groups showed a preference for bodies relative to the object. However, only women with OW preferred their own relative to the control body during intentional processing.

**Conclusion:**

Taken together, results point towards a self-focused and deficit-oriented gaze pattern in women with overweight and obesity. Targeting these processes might help to improve obesity treatment outcomes.

**Level of evidence:**

Level I, experimental study.

**Supplementary Information:**

The online version contains supplementary material available at 10.1007/s40519-022-01419-1.

## Introduction

Body dissatisfaction is markedly increased in individuals with overweight and obesity compared to normal weight persons [1]. Besides negative consequences on emotional well-being, numerous studies confirm the importance of body dissatisfaction for the etiology and maintenance of overweight which is why the integration of body image interventions in obesity treatments is discussed [2–4]. However, to effectively improve body dissatisfaction, a thorough understanding of the underlying mechanisms is warranted.

According to cognitive–behavioral theories, increased body dissatisfaction results from and is maintained by dysfunctional body-related schemata, which distort different mental processes like, e.g., memory, interpretation, and attention [5]. Therefore, two attentional biases have consistently been reported in individuals with eating disorders or high body dissatisfaction: (1) a preferential processing of one’s own compared to a concurrently presented control body and (2) a negative bias towards subjectively disliked body parts [6, 7].

However, only few studies have addressed these biases in overweight and obesity. Only two studies so far have confirmed the preferential processing of one’s own body in obesity during intentional processing [8, 9]. Processing of the pictures were manipulated by either giving information on which side which stimulus would appear (intentional/top–down) or not (bottom–up; [8, 9]). Yet, in both studies, sample characteristics have to be criticized. In one of the two studies, a normal weight-control group is missing to determine the clinical relevance of this bias in obesity [8]. The other study did include a normal-weight control group, but the sample of women with overweight and obesity consisted of a relatively body satisfied population [9]. Thus, it is unclear how the results found translate in a treatment-seeking sample population. Furthermore, it is unclear if the hypervigilance towards one’s own body results from an increased preference of the self or an avoidance of the control body. If the effect is driven by a prioritized processing of one’s own body, the latter should be preferred irrespective of whether a control body or object is present; if avoidance of the control body causes the hypervigilance, this avoidance should also be present during pairings with a control object.

Concerning selective visual attention, three studies confirm the reported deficit-oriented gaze pattern in overweight and obesity [9–11], while another reported an attentional bias towards attractive body parts [12]. These contradicting results are difficult to integrate due to methodological differences (e.g., different definition of fixation duration). Furthermore, significant sample characteristics like body dissatisfaction are missing which might, however, be crucial for subtyping individuals with overweight [13]. Hence, the evaluation of body-related attentional biases among those seeking treatment for body image is especially warranted.

Taken together, evidence concerning attentional biases in individuals with overweight leaves several issues unanswered. Thus, the present study investigates attention allocation to specific body parts as well as attentional processing of concurrently presented (body) stimuli in women with overweight and obesity (OW) seeking treatment for body dissatisfaction compared to women with normal weight (NW). It is hypothesized that, relative to women with NW, treatment-seeking women with OW display a stronger attentional bias towards the self-rated ugliest compared to the most beautiful body part. For the second paradigm, we hypothesized that only during intentional processing, women with OW would show a preferred attention allocation towards one’s own body compared to the control body and object relative to women with NW. Furthermore, significant positive correlations between these attentional biases and state body dissatisfaction were hypothesized.

## Materials and methods

### Participants

The present study was approved by the local ethics committee [614/2015BO2]. Inclusion criteria were (a) 18 ≤ age ≤ 69 years, (b) female gender, (c) corrected/normal vision, (d) fluent in German, and (e) no diagnosis of an eating disorder. Women with OW had to have a body mass index (BMI) of ≥ 25 and women with NW between 18.5 ≤ BMI < 25 [14]. Exclusion criteria were (a) current presence of psychosis, suicidal ideation, manic episode, substance abuse, or irregular intake of antidepressants, (b) pregnancy/lactation period, (c) borderline personality disorder, and (d) participation in eating- and weight-related interventions.

An a-priori calculated power analysis (*f* = 0.035, α = 0.05, power of 0.90) resulted in a total sample size of *n* = 88 (44/group). *N* = 82 women with OW and *n* = 44 women with NW participated in the study.^1^ The presented results are part of a baseline assessment prior to an RCT on body image interventions (results will be presented elsewhere). The groups did not differ on age and marital status; however, education level was lower in OW (e.g., [15]). As expected, women with OW had higher scores on psychopathological variables (see Table 1).

**Table 1:** Descriptive characteristics for women with overweight and obesity (OW) and normal weight controls (NW)

	OW [*n* = 82]	NW [*n* = 44]	Statistics
	Frequency, *n* [%]	Frequency, *n* [%]	
Education level^a^			*χ*^*2*^ [1] = 4.707*
Low High	45 [54.9%] 37 [45.1%]	14 [31.8%] 27 [63.6%]	
Marital status^a^			*χ*^*2*^ [2] = 1.451
With partner Single Widowed/divorced	49 [59.8%] 26 [31.7%] 7 [8.5%]	29 [65.9%] 9 [20.5%] 4 [9.1%]	
SCID I diagnosis			*χ*^*2*^ [1] = 6.140*
No Yes	45 [54.9%] 37 [45.1%]	34 [77.3%] 10 [22.7%]	
SCID II diagnosis^b^			*χ*^*2*^ [1] = 14.309**
No Yes	57 [69.5%] 21 [25.6%]	44 [100%] 0 [0%]	

	*M* [SD]	*M* [SD]	
Age [years]	40.7 [15.9]	37.9 [9.7]	*F*[1, 124] = 1.166
BMI	31.7 [4.7]	21.8 [1.6]	*F*[1, 124] = 185.518**
BDI	9.6 [8.1]	2.4 [4.4]	*F*[1, 124] = 30.270**
EDE_global_	1.7 [0.8]	0.2 [0.3]	*F*[1, 124] = 155.649**
BSQ	103.0 [27.8]	47.2 [15.5]	*F*[1, 124] = 151.635**

	OW [*n* = 79]	NW [*n* = 44]
	*M* [SD]	*M* [SD]
Paradigm 1—questionnaire data		
PANASneg^c^		
*t*0	1.39 [0.40]	1.27 [0.34]
*t*1	1.92 [0.80]	1.17 [0.24]
BISS		
*t*0	3.60 [1.29]	6.38 [1.10]
*t*1	2.57 [1.51]	6.10 [1.33]
Perceived beauty—SB	2.83 [0.69]	4.53 [0.82]
Perceived beauty—CB	3.42 [0.79]	4.55 [0.79]

Paradigm 2—frequency of first fixations^d^				
	Self-body	Vase	Self-body	Vase
Cue condition	5.9 [1.2]	2.1 [1.2]	5.9 [1.4]	2.1 [1.4]
No-cue condition	5.8 [1.3]	2.2 [1.3]	5.9 [1.3]	2.0 [1.3]

	Control body	Vase	Control body	Vase
Cue condition	5.8 [1.4]	2.2 [1.4]	5.7 [1.4]	2.2 [1.3]
No-cue condition	5.7 [1.3]	2.3 [1.3]	5.9 [1.4]	2.0 [1.3]

*BDI* Beck Depression Inventory; *BMI* body mass index; *BSQ* Body Shape Questionnaire; *BISS* Body Image State Scale; *CB* control body; *EDE* Eating Disorder Examination; *educational level: low* ≤ secondary school; *high* baccalaureate or university degree. *PANASneg* negative affect subscale of the Positive and Negative Affect Scale; *SB* self-body; *t0* prior to Paradigm 1, *t1* after Paradigm 1

*p < 0.05; **p < 0.001

^a^*n* = 42 in the NW group due to missing data

^b^*n* = 78 in the OW group due to missing data

^c^*n* = 76 women with OW and *n* = 43 women with NW due to outlier analysis

^d^*n* = 76 women with OW [resp. *n* = 75 for combination self-body—vase] due to outlier analysis

### Stimuli

Digitized black-and-white photos of one’s own body (self-body) and a BMI- and waist-to-hip-matched control body were used. Photos were taken in standardized positions (front, back, left side, right side; face omitted) in standardized underwear (nude panty and top). For the second paradigm, a drawing of an inanimate object (vase) served as control stimulus [16].

### Eye tracking paradigms

Eye movements were measured using a desktop-mounted, video-based infrared remote eye-tracking system (RED250; angular resolution: ≤ 0.5°; temporal resolution: 250 Hz) equipped with iViewXTM2 software (SensoMotoric Instruments).

After a nine-point calibration to ensure gaze accuracy, pictures of the self-body and control body were presented in a free-viewing task with two blocks each including 16 trials in the first paradigm. In each block, every perspective of every stimulus was presented twice (left/right side) for 8 s.

**Fig. 1:**
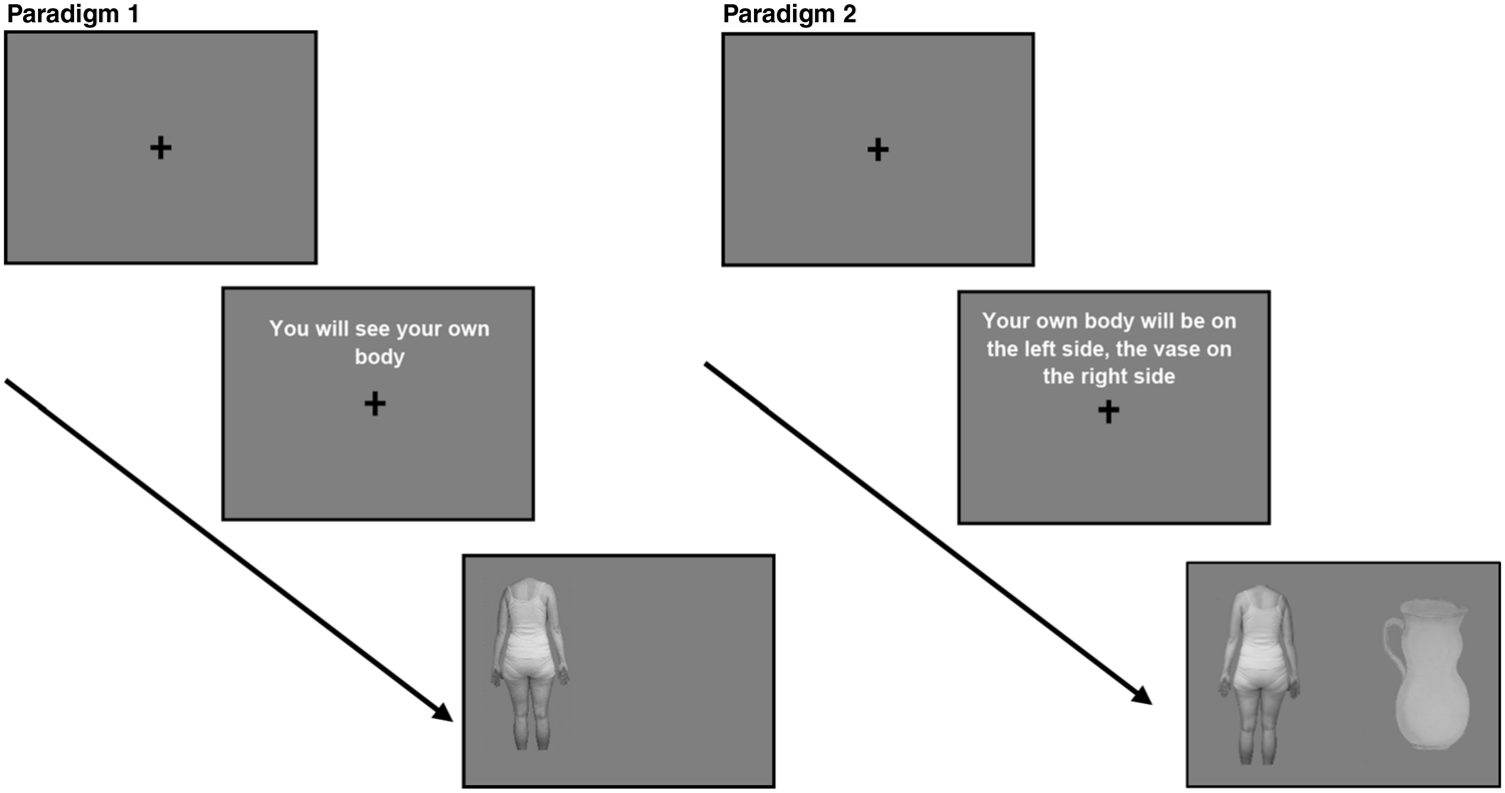
Schematic illustration of the eye-tracking experiments

The second paradigm consisted of two blocks each including 48 trials à 3 s with 16 trials of each picture pair per block with counterbalanced stimulus location (self-body vs. control body; self-body vs. vase; control body vs. vase). Half of the trials were cued with information on which side which stimulus would appear (e.g., “Your own body will be on the right side, the vase on the left side”), while the other half was not cued (e.g., “You will see your own body and the vase”; see Fig. 1).

### Questionnaires and interviews

(1) Body dissatisfaction was assessed using the Body Shape Questionnaire (BSQ; [17]) with higher scores reflecting higher body dissatisfaction (Cronbach’s *α* = 0.98 in the present sample]). (2) The Beck's Depression Inventory (BDI-II; [18]) was used to assess depressive symptoms over the last 2 weeks (*α* = 0.91). (3) The Positive and Negative Affect Scale (PANAS; [19]) was used to assess negative affect prior to and after the first paradigm (*α* = 0.85 prior to [*t*0] and *α* = 0.92 after the experiment [*t*1]). (4) The Body Image State Scale (BISS; [20]) assesses the evaluative experience of one’s own body with higher scores reflecting more favorable states (*α* = 0.90 [*t*0]; *α* = 0.96 [*t*1]). (5) After the paradigms, participants saw printed versions of the self-body and control body and indicated the perceived beauty of ten specified body parts (shoulders, cleavage, arms, hands, breast, back, buttocks, stomach, hips, and thighs) on a 6-point Likert sale (1 = ugly; 6 = beautiful) to calculate a mean score for the overall beauty. Furthermore, participants self-reported their subjectively rated most beautiful and ugliest body part for both bodies (see Supplementary Material for more details).

### Procedure

Participants were recruited via announcements in the local press, flyers, and the university’s mailing list. Trained psychologists performed a diagnostic assessment including the Eating Disorder Examination Interview (EDE; [21]) and the Structured Clinical Interview for Mental Disorders (SCID, [22]).

Informed written consent was obtained, and relevant questionnaires (BSQ, BDI-II) were filled in via Unipark. On a separate day, body pictures were taken. Next, an appointment for the two eye-tracking paradigms was scheduled. Prior to and after the first paradigm, participants filled in the PANAS and BISS. At the end, participants saw printed versions of the self-body and control body to rate and rank the above-mentioned body parts. Afterwards, women with NW were reimbursed and debriefed concerning the study’s rationale,^2^ while another appointment was scheduled with women with OW participating in the RCT.

### Data processing

A fixation was defined as maintaining visual gaze for ≥ 100 ms [23]. For the first paradigm, areas of interests (AOIs) were defined in BeGaze 3.7 (SMI) separately for each participant (ugliest/most beautiful body part). Relative fixation time on the relevant AOI compared to the overall fixation time on the body of all trials including this AOI were calculated. For the second paradigm, the frequency of the direction of the first fixation was analyzed.

### Statistical analysis

Statistical analyses were made using IBM SPSS (Version 26). Mean substitution was used for missing questionnaire data (*n* = 2 for each group). *N* = 3 women with OW dropped out after diagnostic assessment. One participant (first paradigm) and three participants (second paradigm) were excluded due to technical problems. Box plot analyses outlined by SPSS detected *n* = 1 outliers for each group for paradigm 1 and *n* = 1 women with OW resp. *n* = 2 women with NW for paradigm 2.

Hypotheses of paradigm 1 were tested by means of a 2 (Group: OW vs. NW) × 2 (Body: self-body vs. control body) × 2 (Body Part: most beautiful vs. ugliest) repeated-measures ANOVA for relative fixation time. A bias score (= fixation time most beautiful/ugliest body part) for each body was used to calculate Pearson product–moment correlations. For Paradigm 2, three 2 (Group: OW vs. NW) × 2 (Stimulus Combination: self-body vs. vase OR control body vs. vase OR self-body vs. control body) × 2 (Cue: cue condition vs. no-cue condition) repeated-measures ANOVAs were used.

Post hoc ANOVAS and t tests with Bonferroni correction for multiple testing were applied. If assumption of sphericity was not met (Mauchly’s sphericity test: *p* < 0.05), Greenhouse–Geisser correction was applied. Effect sizes of the ANOVAs are reported by partial eta squared (small: *η*_p_^2^ = 0.01; moderate: *η*_*p*_^2^ = 0.06; large: *η*_*p*_^2^ = 0.14) and for *t* tests by Cohen’s *d* (small: *d* = 0.02; moderate: *d* = 0.5 large: *d* = 0.8; [24]).

## Results

### Paradigm 1

#### Relative fixation times

The 2 (Group: OW vs. NW) × 2 (Body: self-body vs. control body) × 2 (Body Part: most beautiful vs. ugliest) ANOVA revealed a significant main effect of Body Part [*F*(1, 118) = 78.026, *p* < 0.001, *η*_*p*_^2^ = 0.398], a significant Group × Body Part interaction [*F*(1, 118) = 28.615, *p* < 0.001, *η*_*p*_^2^ = 0.195] and a significant Body Part × Body interaction [*F*(1, 118) = 35.571, *p* < 0.001, *η*_*p*_^2^ = 0.232]. All other effects were not significant [all *F*s(1, 118) ≤ 1.734, *p*s ≥ 0.191, *η*_*p*_^2^s ≤ 0.14].

**Fig. 2:**
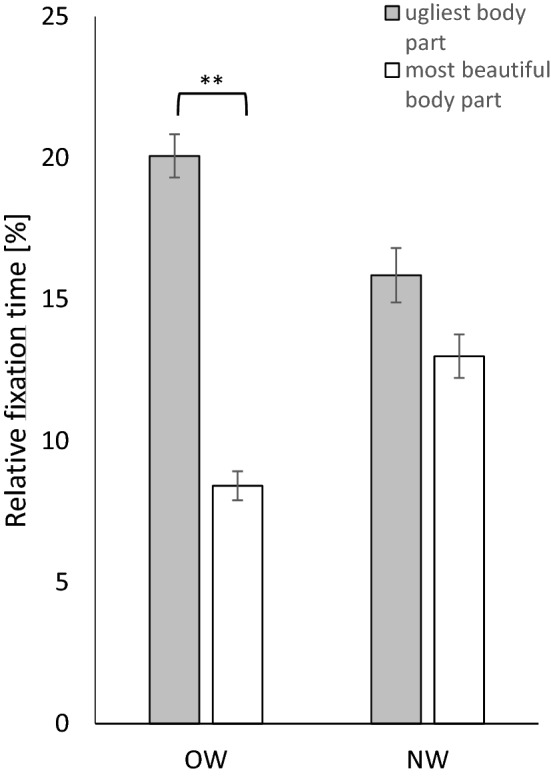
Mean and standard error for the relative fixation time on the subjectively rated ugliest and most beautiful body part in women with overweight and obesity (OW) and normal weight (NW)

Post hoc t tests separated for Group (see Fig. 2) showed that women with OW spent more time looking at the ugliest compared to the most beautiful body part [*t*(76) = 11.913, *p* < 0.001, *d* = 1.33], while women with NW showed a balanced viewing pattern [*t*(42) = 2.153, *p* = 0.074, *d* = 0.296; see Fig. 2].

### Questionnaires

For the PANAS, a significant main effect of Group [*F*(1, 117) = 25.604 *p* < 0.001, *η*_*p*_^2^ = 0.180], Time [*F*(1, 117) = 15.192, *p* < 0.001, *η*_*p*_^2^ = 0.115] and a significant interaction of Group × Time [*F*(1, 117) = 34.148, *p* < 0.001, *η*_*p*_^2^ = 0.226] were found. Post hoc t tests showed a decrease in negative affect in women with NW [*t*(42) = 3.479, *p* = 0.001, *d* = 0.639] and an increase in women with OW [*t*(75) = − 6.635, *p* < 0.001, *d* = 1.315].

The same pattern was found for the BISS [Group: *F*(1, 121) = 176.630, *p* < 0.001, *η*_*p*_^2^ = 0.593; Time: *F*(1, 121) = 57.840, *p* < 0.001, *η*_*p*_^2^ = 0.323; Group × Time: *F*(1, 121) = 15.462, *p* < 0.001, *η*_*p*_^*2*^ = 0.113]. Post hoc *t* tests revealed a significant decrease in body satisfaction over time, albeit stronger in women with OW [OW: *t*(78) = 8.982, *p* < 0.001, *d* = 1.121; NW: *t*(43) = 2.697, *p* = 0.020, *d* = 0.464].

### Correlations

#### Paradigm 1

The bias score for the self-body was significantly and positively correlated with the beauty rating of the self-body (*r* = 0.432; *p* < 0.001), but not the control body (*r* = 0.172; *p* = 0.066). Significant positive correlations between changes in state body satisfaction and the bias score of the self-body (*r* = 0.191; *p* = 0.042) and the control body (*r* = 0.223; *p* = 0.017) were found. The same result pattern emerged for changes in mood (self-body: *r* = 0.276; *p* = 0.003; control body: *r* = 0.224; *p* = 0.017).

### Paradigm 2

#### Self-body vs. vase

The 2 (Group: OW vs. NW) × 2 (Stimulus Combination: self-body vs. vase) × 2 (Cue: cue condition vs. no-cue condition) ANOVA for the frequency of the first fixation revealed only a significant main effect of Stimulus [*F*(1, 117) = 287.801, *p* < 0.001, *η*_*p*_^2^ = 0.711; all other *F*s(1, 117) ≤ 2.220, *p*s ≥ 0.139, *η*_*p*_^2^s ≤ 0.019]. More first fixations were directed towards the self-body compared to the object.

#### Control body vs. vase

The same result pattern was found for this combination [Stimulus Combination: *F*(1, 118) = 255.831, *p* < 0.001, *η*_*p*_^2^ = 0.684; all other *F*s(1, 118) ≤ 1.745, *p*s ≥ 0.189, *η*_*p*_^2^s ≤ 0.015].

#### Self-body vs. control body

**Fig. 3:**
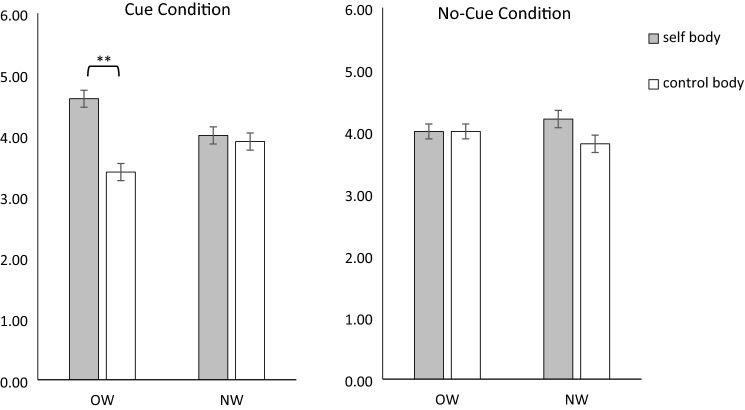
Mean and standard error for the frequency of first fixations on the self-body and the control body in women with overweight and obesity (OW) and normal weight controls (NW)

A significant main effect of Stimulus [*F*(1, 116) = 7.549, *p* = 0.007, *η*_*p*_^2^ = 0.061] and a significant Group × Stimulus Combination × Cue  interaction were found [*F*(1, 116) = 7.274, *p* = 0.008, *η*_*p*_^2^ = 0.059; all other *F*s(1, 116) ≤ 3.556, *p*s ≥ 0.062, *η*_*p*_^2^s ≤ 0.030]. Post hoc ANOVAs separated for Group revealed no significant effects for women with NW [all *F*s(1, 41) ≤ 1.250, *p*s ≥ 0.270, *η*_*p*_^2^s ≤ 0.030]. For women with OW, there was no main effect of Cue [*F*(1, 75) = 2.626, *p* = 0.109, *η*_*p*_^2^ = 0.034], but a significant main effect of Stimulus Combination [*F*(1, 75) = 9.652, *p* = 0.003, *η*_*p*_^*2*^ = 0.114] and a Stimulus Combination × Cue interaction [*F*(1, 75) = 13.383, *p* < 0.001, *η*_*p*_^2^ = 0.151]. Follow-up t tests revealed a higher number of first fixations towards the self-body compared to the control body [*t*(75) = 4.363, *p* < 0.001, *d* = 0.500] in the cue condition, but not in the no-cue condition [*t*(75) = 0.083, *p* = 0.934, *d* = 0.010; see Fig. 3].

### Correlations

#### Paradigm 2

There was a negative correlation between the fixation frequency on the self-body in the cue condition and the perceived beauty rating of the self-body (*r* = − 0.204; *p* = 0.027), but not with the control body (*r* = − 0.143; *p* = 0.123).

#### Correlations with BMI

In the group with women with OW, there were neither significant correlations between BMI and questionnaire data (body dissatisfaction [BSQ; *r* = − 0.011, *p* = 0.924]; depressive symptoms [BDI-II; *r* = − 0.029, *p* = 0.797], changes in state body dissatisfaction and negative affect during the experimental tasks [BISS_*t*0–*t*1)_; *r* = − 0.052, *p* = 0.648; PANAS_neg *t*0–*t*1_; *r* = − 0.216, *p* = 0.056] nor with the experimental viewing patterns (*r* ≤|0.175|, *p* ≥ 0.131).

## Discussion

As biased information processing is theorized to be involved in the maintenance of body dissatisfaction [5], the aim of the present study was to investigate attentional processing of body pictures in women with overweight and obesity without an eating disorder compared to women with normal weight.

As hypothesized, women with overweight and obesity displayed a negative bias towards the ugliest compared to the most beautiful body part, whereas women with normal weight showed a balanced viewing pattern. This is in line with previous studies in overweight and obesity [9–11] and in eating disorders [6, 7]. The negative attentional bias has been related to body dissatisfaction [25], eating pathology [26], and higher BMI and lower attractiveness ratings of one’s own body [27] in previous studies; all of these variables were significantly higher in the OW compared to the NW group in the present study. Interestingly, women with overweight and obesity not only explored their own, but also the control body in a deficit-oriented manner, which replicates findings of a recent study [28]. In this study, this deficit-oriented viewing pattern has been interpreted as reflecting the negative cultural views on obesity. Importantly, these negative attitudes towards overweight and obesity are also shared by the overweight community itself [29], and are associated with poor mental health and higher body dissatisfaction [30]. Hence, the deficit-oriented viewing pattern found in the present study might contribute to the maintenance of these dysfunctional internalized attitudes [5].

Concerning the second paradigm, initial attentional orientation differed significantly between the groups: while participants with normal weight equally distributed their first fixation towards both bodies, women with overweight and obesity showed a preferred attention allocation towards their own body, but only during intentional processing thereby replicating previous findings from a non-treatment-seeking overweight sample [9]. As in both groups, participants’ attention was captured by the body pictures in trials including the object, we can interpret this finding as a prioritized processing of one’s own rather than an avoidance of the control body. Hence, the hypervigilance found for one’s own body is of clinical relevance and presumably results from top–down processes involving the activation of underlying negative body-related self-schemata. This finding corroborates studies from eating disorder research [6] and a recent study in women with high body image concerns [31]. The latter found a significant correlation between the hypervigilance towards one’s own body and the tendency to evaluate the self-body as less attractive than a control body [31]. This self-deprecating discrepancy was also evident in the present sample. As the beauty rating of the self-body correlated significantly with the preferred processing of one’s own body, these variables might contribute to the emotional relevance of one’s own body for women with overweight and obesity [32, 33].

To sum up, the present study provides evidence that women with overweight and obesity preferentially attend to and explore their own body in a deficit-oriented manner. As individuals with overweight and obesity show a reduced disengagement from obese compared to thin bodies [34], initial orientation towards one’s own body might start a vicious circle of deficit-oriented attention towards one’s own body. According to cognitive theories [5], this might contribute to the maintenance of negative body-related schemata. This assumption is supported by the self-reported deteriorations in negative mood and state body dissatisfaction following the paradigms and the significant, albeit small correlations of these variables with the deficit-oriented attentional bias. As first experimental studies confirm the causal link between attentional biases and body dissatisfaction [35], future studies should examine whether these dysfunctional attentional biases are modifiable and whether such modifications improve body satisfaction in obesity.

## Strengths and limitations

The present study was able to provide a thorough insight into attentional processing of body pictures in women with overweight and obesity using an experimental approach and addressing limitation of previous studies (e.g., non-body control stimulus, normal weight controls, treatment-seeking sample of women with overweight and obesity, etc.). However, there are several limitations. First, a counterbalanced approach should be used, as familiarity with the pictures might have influenced results. Second, the external validity of our stimuli is limited as only static black-and-white pictures were used. Future studies should include colorful pictures or the confrontation with one’s own body in virtual reality or in vivo [36, 37]. Third, during the second paradigm, we tried to disentangle automatic and intentional processing of body images by giving information about the localization. Even though, we were able to show differences in processing, other paradigms including one’s own body as task-irrelevant stimulus (e.g., visual probe task) might be more suitable to tap into bottom–up processing. Our last limitation concerns the heterogeneous BMI in the OW group within our relatively small sample size. Notably though, there were no significant correlations between BMI and psychopathological variables as well as the observed viewing patterns. This indicates that the results were not driven by specific BMI subgroups. Nonetheless, future studies should compare different BMI classes in overweight and obesity in regard to the assessed variables, as previous studies have found associations between BMI and psychological variables, like, e.g., body dissatisfaction [38] as well as dysfunctional viewing patterns [27].

To sum up, our results point towards a self-focused, deficit-oriented exploration of one’s own body in women with overweight and obesity without an eating disorder that might contribute to the maintenance of body dissatisfaction. As body dissatisfaction negatively influences psychological well-being and weight-loss success [39], targeting these attentional biases might help to enhance obesity treatment outcomes.

## What is already known about this subject?

- Attentional biases play an important role in the maintenance of body dissatisfaction
- A few studies have adressed this issue in overweight and obesity despite the negative impact body dissatisfaction has on psychological well-being and weight-loss success in obesity

## What this study adds?

- Specification of attentional processes underlying body dissatisfaction in overweight and obesity using experimental eye-trakcing paradigms and adressing limitations of previous studies [e.g., including an inanimate control object]
- Demonstrating the clinical relevance of these attentional biases by including a control group.
- The identified processes might be used in interventions to improve body dissatisfaction.

## Supplementary Information

Below is the link to the electronic supplementary material.

Supplementary file1 (DOCX 13 KB)

## Footnotes

1 Note: The presented unbalanced sample size is due to the accompanying RCT. Homogeneity of variance was therefore tested and, if not given, non-parametric tests were applied [see Statistics].

2 Prior to testing, participants were told a cover story [assessment of changes in pupil dilatation as an indicator of emotional arousal].
