# Supplementary material for: Attentional processing of body images in women with overweight and obesity
Source: Eat Weight Disord. 2022 Jul 4;27(7):2811–9. doi: 10.1007/s40519-022-01419-1 (PMC9556367; doi:10.1007/s40519-022-01419-1)
Supplement: Supplementary file 1 — Supplementary file1 (DOCX 13 KB) [file 40519_2022_1419_MOESM1_ESM.docx]

**Supplementary Material**

**Results**

**Identification of the ugliest/most beautiful body part:** Women with OW rated cleavage most frequently as most beautiful and stomach as ugliest body part of both bodies. Women with NW reported breast to be the most beautiful and thighs to be the ugliest body part of the self-body, whereas for the control body, thighs were rated as most beautiful and buttocks/hands as ugliest part.

**Perceived beauty:** The 2 (Group: OW vs. NW) ×2 (Body: self-body vs. control body) ANOVA showed a significant main effect of Group (*F*(1,121) = 154.157, *p <* .001, *η_p_²* = .560) and Body (*F*(1,121) = 12.294, *p =* .001, *η_p_²* = .092) and a significant interaction Group × Body (*F*(1,121) = 10.664, *p =* .001, *η_p_²* = .081). While women with NW rated both bodies similarly (*t*(43) = 0.163, *p=* .871. *d* = 0.024), women with OW rated the self-body significantly less beautiful than the control body (*t*(78) = 5.435, *p* < .001, *d* = 0.660).
